# Revisiting the sialome of the cat flea *Ctenocephalides felis*

**DOI:** 10.1371/journal.pone.0279070

**Published:** 2023-01-17

**Authors:** Stephen Lu, Monika Danchenko, Kevin R. Macaluso, José M. C. Ribeiro

**Affiliations:** 1 Laboratory of Malaria and Vector Research, National Institute of Allergy and Infectious Diseases, National Institutes of Health, Bethesda, Rockville, Maryland, United States of America; 2 Department of Microbiology and Immunology, University of South Alabama College of Medicine, Mobile, Alabama, United States of America; University of Cincinnati, UNITED STATES

## Abstract

The hematophagous behaviour emerged independently in several instances during arthropod evolution. Survey of salivary gland and saliva composition and its pharmacological activity led to the conclusion that blood-feeding arthropods evolved a distinct salivary mixture that can interfere with host defensive response, thus facilitating blood acquisition and pathogen transmission. The cat flea, *Ctenocephalides felis*, is the major vector of several pathogens, including *Rickettsia typhi*, *Rickettsia felis* and *Bartonella* spp. and therefore, represents an important insect species from the medical and veterinary perspectives. Previously, a Sanger-based sialome of adult *C*. *felis* female salivary glands was published and reported 1,840 expressing sequence tags (ESTs) which were assembled into 896 contigs. Here, we provide a deeper insight into *C*. *felis* salivary gland composition using an Illumina-based sequencing approach. In the current dataset, we report 8,892 coding sequences (CDS) classified into 27 functional classes, which were assembled from 42,754,615 reads. Moreover, we paired our RNAseq data with a mass spectrometry analysis using the translated transcripts as a reference, confirming the presence of several putative secreted protein families in the cat flea salivary gland homogenates. Both transcriptomic and proteomic approaches confirmed that FS-H-like proteins and acid phosphatases lacking their putative catalytic residues are the two most abundant salivary proteins families of *C*. *felis* and are potentially related to blood acquisition. We also report several novel sequences similar to apyrases, odorant binding proteins, antigen 5, cholinesterases, proteases, and proteases inhibitors, in addition to putative novel sequences that presented low or no sequence identity to previously deposited sequences. Together, the data represents an extended reference for the identification and characterization of the pharmacological activity present in *C*. *felis* salivary glands.

## Introduction

Fleas are small, hematophagous insects classified in the order Siphonaptera that contains over 2,500 known species that can parasitize a wide range of mammals and birds [1, 2]. Fleas live in close association with their hosts and have evolved unique morphological traits to assist their survival. Their laterally flat body facilitates their movement through the host fur and feathers, and they also possess ctenidia, sclerotized setae on their body and legs that aid in preventing their unwanted dislodgment from the host. Other key adaptations include the ability to jump over great distances and, as adults, the presence of specialized mouthparts that facilitate piercing of the host skin [2].

From a medical perspective, fleas have shaped human history as vectors of the Gram-negative bacterium *Yersinia pestis*, the causative agent of the bubonic plague, that substantially reduced the human population in the past [3, 4] and continues to be a burden today as cases are still reported in Africa, USA, South America and Asia [5, 6]. Additionally, fleas are also implicated in the transmission of other pathogens, such as *Rickettsia typhi* (murine fever), *Bartonella henselae* (cat-scratch disease) and *Rickettsia felis* (flea-borne spotted fever) [7, 8], taxing human health. Because fleas live in close association with their host and can efficiently feed on a wide variety of vertebrates [9], they pose a significant threat to companion animals and, therefore, are also relevant from veterinary and economic perspectives [10].

Blood acquisition is a pharmacological endeavour for any hematophagous arthropod. To succeed, the blood feeder must overcome a plethora of defensive mechanisms deployed by its host. Upon piercing the host skin, a complex signalling pathway will trigger the coagulation cascade, platelet activation, vasoconstriction as well as inflammatory and antimicrobial responses. The deep RNA sequencing of salivary glands paired with the structural and functional characterization of salivary proteins established that hematophagous arthropods convergently evolved different mixtures of salivary proteins (sialomes) that can modulate and interfere with the host responses [11]. In addition to facilitating blood acquisition, it was also demonstrated that vector saliva can enhance pathogen transmission [11–14], inspiring the use of salivary proteins for vaccine development [15].

Despite its importance, the pharmacological activity of flea saliva has been understudied when compared to other hematophagous vectors (e.g., ticks, mosquitoes, sand flies and triatomines) and, so far, only a few molecules have been functionally characterized [16–19]. Additionally, although the *C*. *felis* genome was recently described [20], there is an overall lack of knowledge regarding the composition of *C*. *felis* salivary glands with the exception of a single Sanger-based sialome [21]. Here we revisit the *C*. *felis* sialome using an Illumina-based approach paired with mass spectrometry analysis of the flea salivary gland homogenates. Together, the data presented here expands the current repertoire of flea salivary proteins and serves as a foundation for further studies aimed at the identification and characterization of the as yet unknown pharmacological activity present in *C*. *felis* salivary glands.

## Materials and methods

### *C*. *felis* salivary gland collection

A population of EL cat fleas (Elward II Laboratory colony, Soquel, CA) was maintained at the University of South Alabama College of Medicine under standardized insectary conditions as previously described [7]. For the collection of flea salivary glands, 200–220 newly emerged, mixed-sex (ratio 1:1) adult fleas were fed defibrinated bovine blood (HemoStat Laboratories, USA) in an artificial feeding system [22]. After feeding for five consecutive days, female fleas were collected and cleaned by sequential surface washing with 10% bleach for 2 min, 70% ethanol for 2 min, followed by rinsing with sterile distilled water three times for 2 min. Fleas were then immobilized on ice, and salivary glands were dissected under a standard stereomicroscope in 0.01 M sterile phosphate-buffered saline (PBS) pH 7.2 (Gibco, USA) on a glass depression slide [23].

For transcriptomic analysis, 188 pairs of intact fed female salivary glands were pooled in RNA*later* solution (Ambion, USA) and incubated at 4°C for two days, then stored at −80°C until further processing. For proteomic analysis, 220 pairs of intact salivary glands were collected in sterile PBS pH 7.2 on ice and frozen at −80°C shortly after microdissection.

### RNA extraction, Illumina sequencing and (data) analysis

Total RNA was extracted from the fed female C. *felis* salivary glands with the RNEasy isolation kit (QIAGEN, USA) according to the manufacturer’s specifications and analyzed with an Agilent 2100 Bioanalyzer (Agilent, USA). The library was constructed using the NEBNextUltraTM II Directional RNA Library Prep Kit and sequencing was performed in an Illumina Novaseq 6000 DNA sequencer (Illumina, USA). The Illumina reads were trimmed of the Illumina adapters and low-quality sequences (Q < 20) using TrimGalore (https://github.com/FelixKrueger/TrimGalore), merged into a single file, and assembled using ABySS (2.3.1) [24] with k values from 25 to 95 (with increments of 10) in single stranded mode and Trinity (2.13.2) [25] in single stranded F mode. The assemblies from ABySS and Trinity were combined and filtered with the CD-HIT tool [26]. Coding DNA sequences (CDS) with open reading frames larger than 150 nucleotides were extracted based on BLASTp results to a subset of the non-redundant protein database and the TSA database and were selected if fragments shared ≥ 70% similarity with a matching protein. Additionally, all open reading frames (ORF’s) starting with methionine and having 40 amino acids in length were submitted to the signalP program (v. 3.0); fragments that possessed a signal peptide were mapped to the ORF’s, and the most 5’ methionine leading to a signal peptide was selected as the starting methionine of the transcript coding for a putative secreted peptide [27]. To assess our assembly quality, the BUSCO (4.1.3) benchmark for universal single-copy orthologs using the Insecta database (2022-04-05) was used [28]. For annotation, we used an in-house program that scans a vocabulary of ~ 400 words and their order of appearance in the protein matches from BLASTp/rpsBLAST results with different databases (TSA, subset of the NR, refseq-invertebrate, refseq-vertebrate, MEROPS, PFAM and CDD), including their e-values and coverage. Relative quantification of each CDS was estimated using the TPM (transcripts per million) parameter by mapping the trimmed library reads to the extracted CDS using the RSEM tool [29], and the final annotated CDS was exported to a hyperlinked Excel spreadsheet.

### Mass spectrometry analysis

For the mass spectrometry analysis, 220 salivary gland pairs from fed *C*. *felis* adult females were pooled and homogenized in PBS pH 7.2 using a sterile pestle (Sigma, USA). The sample was centrifuged (10,000 ×*g*, for 10 min at 4°C), the supernatant collected, and the total protein concentration determined using a BCA protein kit (PIERCE, USA) according to the manufacturer’s instructions. An aliquot of the salivary gland homogenate containing 3 μg of total protein was diluted in 50 mM HEPES pH 8.0 to a final volume of 30 μl. The protein was reduced with 5 mM dithiothreitol for 40 min at 37°C. The samples were cooled to room temperature and alkylated with 15 mM iodoacetamide for 20 min. Then, 200 ng of trypsin were added, and samples were incubated for 15 h at 37°C. The pH was adjusted to approximately 2.5 with 10% trifluoroacetic acid (TFA) and samples were desalted and concentrated with Agilent OMIX10 tips (Agilent, USA). Peptides were eluted with 20 μl of 0.1% TFA/50% acetonitrile (ACN) and dried under vacuum. The peptides were dissolved in 12 μl of 0.1% formic acid (FA)/3% ACN and centrifuged at 18,000 × *g* for 5 min. The LC-MS experiment was performed using Orbitrap Fusion Lumos mass spectrometer (Thermo Fisher Scientific, USA) coupled to EASY nLC 1200 nano-liquid chromatography system (Thermo Fisher Scientific, USA). Peptides were first bound to a PepMap C18 column (3 μm particle, 100 Å pore, 75 μm inner diameter, 2 cm length) then separated using an EASY-Spray analytical column (PepMap C18, 2 μm particle, 100 Å pore, 75 μm inner diameter, 25 cm length) using a linear gradient of 0 to 40% ACN containing 0.1% FA for 100 min, followed by 40–80% for 5 min, 80% hold for 5 min, 80–0% for 5 min, and 0% hold for 5 min. Data were acquired with a standard data-dependent acquisition strategy, in which the survey MS1 scan was done at least every 3 sec with the Orbitrap mass analyzer at 120,000 resolution. The MS2 scans were done with a linear ion trap mass analyzer for multiply charged precursor ions isolated with a 1.6 m/z window using a quadrupole and fragmented by CID at 35% collision energy. The EASY-IC internal calibration was utilized for Orbitrap scans, and the dynamic exclusion period was set at 60 sec. Tandem mass spectra were analyzed using the PatternLab for proteomics 4.0 platform [30]. A target-decoy database was prepared using the CDS obtained from the RNAseq analysis and searched using the Comet tool [31] implemented in PatternLab. The search space included all semi-tryptic peptide candidates and carbamidomethylation of cysteine was used as a static modification. Data were searched with a 50 ppm precursor ion tolerance and a 0.4 Da fragment ion tolerance. The validity of the peptide spectrum matches (PSMs) generated by Comet was assessed using the Search Engine Processor (SEPro) module from PatternLab. A cutoff score was established to accept a protein false discovery rate (FDR) of 1% based on the number of decoys. Results were post-processed to only accept PSMs with <10 ppm precursor mass error, and only proteins with at least one unique peptide were considered. The normalized spectral abundance factor (NSAF) was used to represent the relative abundance of proteins.

### Statistical analysis

The Spearman correlation between proteomic and transcriptomic relative quantifications was evaluated using the Log_2_ (NSAF x 10^4^) and the Log_2_ TPM. The scatterplot and determination of the Spearman correlation coefficient (Rho) were calculared using the R programming language [32] with the *stats* package. Phylogenetic trees were constructed using the maximum likelihood method [33] with MEGA X [34], while the amino acid alignments were done with the Clustal Omega tool [35].

### Data availability

The transcriptome data was deposited to the National Center for Biotechnology Information (NCBI) under Bioproject PRJNA850944 and Biosample accession SAMN29207207. The raw reads were deposited to the Short Reads Archive of the NCBI under accession SRR19752890, and the CDS deposited to the Transcriptome Shotgun Assembly (TSA) under accession GKAT00000000. The raw proteomic data was deposited to the ProteomeXchange platform under accession number PDX034807.

## Results and discussion

### Overall description of *C*. *felis* sialotranscriptome and sialoproteome

Illumina-based RNA-sequencing of *C*. *felis* salivary glands resulted in 42,754,615 high quality reads. Our *de novo* assembly strategy produced 85,669 sequences from which 11,752 CDS were extracted based on their homology with previously deposited sequences or by the presence of a putative signal peptide. To assess the quality of our assembly, we performed the BUSCO analysis of completeness using the Insecta database as a reference. The current dataset displayed a completeness of 80.7% (76.5% single and 4.2% duplicated), 2.9% of fragmentation and 16.4% of missing sequences, which is within the range of previous genomic and transcriptome assemblies [36–38]. For comparison, we conducted the same analysis using the *C*. *felis* genome assembly [20] and observed a completeness of 94% (49.5% single and 44.5% duplicated) with 0.5% of fragmented and 5.5% of missing sequences. Together, these analyses suggest no major bias in our assembly pipeline.

To estimate each CDS abundance, we mapped the trimmed reads to the extracted CDS using the RSEM tool. This resulted in an alignment of 39.5% of all reads; similar results were reported in previous sialome studies [37, 39, 40]. The remaining unmapped reads are potentially from the 5`and 3`UTR of the CDS, from non-coding RNA, or any sequence that failed to be extracted by not generating an ORF with at least 150 nucleotides, lacked a putative signal peptide, or failed to produce matches against previously deposited proteins.

For the functional annotation of the extracted CDS, we selected sequences with at least 150 nucleotides and TPM higher than one, resulting in 8,892 sequences. The annotated CDS were exported to a hyperlinked Excel spreadsheet that is currently available for download (S1 File). The annotated CDS were used as a reference database for the proteomic part of the study resulting in the identification of unique peptides from 902 proteins (S2 File). As observed in other sialomes from blood-feeding arthropods [39–41], the “secreted” class was the most abundant functional class in both transcriptomic and proteomic relative quantifications (Table 1), with 519 CDS accounting for 45.5% of all TPM and 107 proteins representing 21.3% of all quantified protein in the salivary gland homogenate. The second most abundant functional group in our transcriptome was the “unknown” group (1,052 sequences representing 13.4% of all transcripts) which comprises novel CDS since they have low or no sequence identity to previously deposited sequences, while the second most abundant functional class in our proteomic analysis was the “protein modification” class with 92 proteins accounting for 12.7% of all identified proteins.

**Table 1:** Functional classification and quantification of transcripts and proteins identified in *C*. *felis* salivary gland homogenates.

Class	Number of Contigs	TPM^a^ (%)	Number of proteins	NSAF^b^ (%)
Cytoskeletal	274	0.42	41	2.81
Extracellular matrix/cell adhesion	112	0.11	12	0.48
Immunity	76	0.09	6	0.32
Metabolism, amino acid	137	4.36	30	2.28
Metabolism, carbohydrate	152	0.29	16	1.05
Metabolism, energy	376	1.72	58	7.65
Metabolism, intermediate	81	0.13	4	0.14
Metabolism, lipid	248	0.35	27	1.29
Metabolism, nucleotide	142	0.2	9	0.28
Nuclear export	36	0.02	2	0.08
Nuclear regulation	342	0.32	19	1.78
Oxidant metabolism/detoxification	69	0.2	15	2.68
Proteasome machinery	305	0.74	39	2.24
Protein export machinery	484	1.23	62	4.06
Protein modification machinery	403	4.39	92	12.72
Protein synthesis machinery	370	11.18	102	11.60
**Secreted**	**519**	**45.52**	**107**	**21.32**
Signal transduction	871	1.69	56	3.57
Storage	20	0.12	6	1.24
Transcription factor	32	0.05		-
Transcription machinery	1,309	1.53	70	4.39
Transporters/storage	276	0.71	21	0.69
Transposable element	206	0.09		
Unknown	1,052	13.37	77	9.39
Unknown, conserved	992	11.17	31	7.94
Viral	8	0.01		
Total	8892		902	

^a^TPM: Transcripts per million

^b^NSAF: Normalized spectral abundance factor

A deeper insight into the “secreted” class revealed a high abundance of CDS similar to the FS-H family [42] with 87 CDS representing ~ 34% of all secreted transcripts. The second most abundant secreted protein families were CDS classified as “unknown” (108 CDS accounting for 33.5% of all secreted proteins), followed by the acid phosphatase family (35 CDS representing 15.6% of all secreted proteins) and the “unknown conserved” (161 CDS representing ~7.9%) group. Similar results were observed in our LC-MS analysis. Unique peptides from 27 members of the FS-H family were identified and, together, they accounted for 34.4% of all secreted proteins. The second most abundant salivary protein family was represented by the acid phosphatases consisting of 21.4% of all secreted proteins. Finally, the proteins classified as “unknown” and “unknown conserved” represented 11.6% and ~13.8% of all secreted proteins, respectively (Table 2). Together these four functional groups represent ~91% of all secreted CDS and 81% of all secreted identified proteins. A very similar composition was observed in the recent rodent flea, *Xenopsylla cheopis*, sialome, in which the acid phosphatases were found to be the most abundant salivary proteins, followed by the FS-H-like protein family [37], indicating an overall conservation of flea sialome composition.

**Table 2:** Relative abundance of protein families found within the ‘secreted’ functional class.

Protein family	Number of CDS	TPM (%)	No. proteins	NSAF (%)
Acid phosphatases	35	15.5812	9	21.37
Alpha macroglobulin	2	0.0044	-	-
Amino peptidase	3	0.0023	-	-
Angiotensin-converting enzyme	1	0.0031	-	-
Antigen 5	15	4.762	8	5.24
Apyrase	10	0.7045	3	2.21
Aspartic peptidase	1	0.0027	1	1.94
Cholinesterase	25	2.3271	7	4.36
Cysteine peptidases	5	0.0116	4	0.78
FS-H	87	33.9826	27	34.42
Hormone binding	4	0.013	-	-
Inhibitor	9	1.1078	4	1.84
Lipases	2	0.0009	-	-
Mucin	6	0.0042	1	0.28
OBP	6	0.0523	2	2.01
Serine peptidases	9	0.0049	1	0.16
Unknown	108	33.5562	13	11.60
Unknown, conserved	161	7.8792	23	13.78

A common caveat in RNA-seq transcriptome studies is the relation between the CDS quantification (TPM or FPKM) and the protein concentration in the sample. To address this question, we determined the Spearman correlation between the Log_2_TPM and the Log_2_NSAF (Rho = 0.5883, p < 2.2x10^-16^, S1 Fig). A similar result was found when we determined the Spearman correlation of proteins belonging to the “secreted” class. The moderate and significant correlation between transcript and protein relative quantifications indicates that transcript levels are a reasonable measure to estimate protein concentration of salivary proteins from the cat flea.

It’s important to note that the chromosome-level assembly of *C*. *felis* genome revealed an unprecedented level of genomic variability between fleas from the same colony, and variations up to 118 Mb were observed between two individuals [20]. This observation has a direct impact on the interpretation of the current data as our samples originated from pooled salivary glands. Additionally, the pooled sample used for the proteomic analysis was different from the one used in our transcriptome, impacting the overall correlation between the proteomic and transcriptomic quantifications. Moreover, the high variability of secreted protein sequences observed in the current study is probably not from a single flea but, instead, scattered among different individuals. Thus, the data presented here represent a common *core* of transcripts and proteins found in the salivary glands of the cat flea. In addition to the abundant FS-H and acid phosphatase families, we also reported here several sequences similar to the antigen 5-like, cholinesterase, apyrase, odorant-binding protein, peptidases, and peptidases inhibitors that were also found in the previous cat and rat flea sialomes [21, 37, 43].

### A deep insight into the core secreted salivary proteins of *C*. *felis*

In the section that follows, we present a discussion of the secreted protein families found in this study, focusing on the protein families identified in the LC-MS analysis and their possible role in blood acquisition.

#### FS-H / FS-I

**Fig 1:**
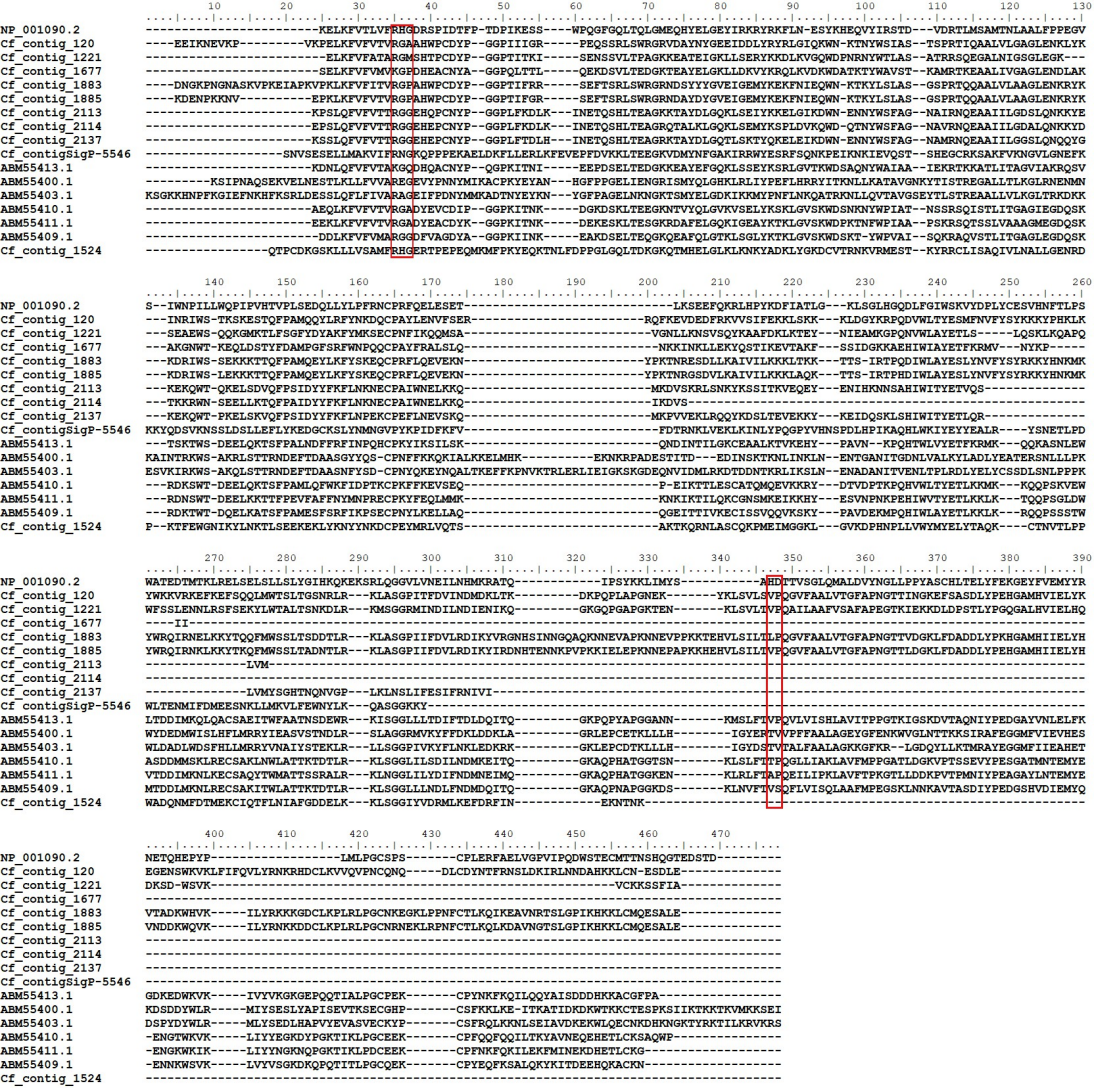
Amino acid alignment of members of the FS-H protein family. Sequences from *C*. *felis* salivary glands (Cf_contig_XXXX) detected by LC-MS analysis with previously deposited identified FS-H members from the rat flea *X*. *cheopis* (Xc_XXX and ABMXXXXX) and scorpion toxins from *Centruroides noxius* (sp|P15223), *Tityus obscurus* (sp| H1ZZI4), *Mesobuthus martensii* (sp|P15228), *Leiurus hebraeus* (sp|P0C5I9) and *Rhopalurus junceus* (sp|E7CLP2). The conserved cysteine residues are shaded in black.

Members of this protein family possess eight cysteine residues, including a C-X-C C-terminus motif (Fig 1), and these were first reported as the major salivary antigen of the cat flea [44, 45]. Interestingly, when comparing sialomes studies, it seems that the FS-H protein family is unique to fleas since they have not been identified in other blood-feeding vectors, such as ticks, flies, mosquitoes or kissing bugs [46]. Yet, a conserved 6-cysteines framework can be found between the FS-H family, insect defensins and scorpion toxins [47], suggesting a similar fold and potential role for the FS-H-like proteins. In the previous *C*. *felis* sialome, several FS-H-like proteins were found and accounted for most of the secreted proteins identified when considering the number of clones sequenced as a quantitative measurement [21]. Corroborating this finding, our transcriptomic and proteomic analyses revealed that the FS-H-like proteins are the most abundant salivary protein family of *C*. *felis*. Here we identified 87 CDS belonging to this protein family with a wide range of TPM values (2.11–58,813) (S1 File) that accounted for almost 34% of all secreted transcripts. Likewise, our LC-MS analysis identified unique peptides from 27 FS-H-like proteins representing 34.4% of all secreted proteins quantified (Table 2).

Two members of this salivary protein family (FS50 and FS48) have been functionally characterized and these were initially identified from the rat flea sialome [43]. Functional and structural studies with FS50 revealed a core structure similar to that of scorpion toxins. Moreover, it was demonstrated that FS50 blocks the Na_V_1.5 sodium channel [17]. Similarly, FS48 was shown to act as an immunomodulator of T cells by blocking the voltage-gated potassium channel K_V_1.3 [18, 48]. Despite the above-mentioned evidence, FS50 and FS48 channel blocking mechanism is still unclear, because the key residues responsible for this activity in the scorpion toxins are absent in the FS-H members. In addition to their channel blocking activity, the FS-H-like proteins also appear to be related to *R*. *felis* infection. A recent study evaluated the temporal expression pattern of four FS-H-like transcripts (Cf-169, Cf-65, Cf-12 and Cf-75) in different time points post-infection [23]. In the current dataset we found that CDS contig_5185 was almost identical (96.3%) to Cf-12 (S2 Fig), and unique peptides from contig_5185 were also found in our LC-MS analysis, supporting the hypothesis that these two proteins are present in *C*. *felis* salivary glands (S2 File). Furthermore, BLASTp of Cf-169, Cf-65 and Cf-75 against the current dataset resulted in the identification of contig_798 (89% identity), contig_4556 (98% identity), contig_8628 (64% identity), respectively. However, none of these sequences were identified in the mass spectrometry analysis.

A comparison of the primary structure of *C*. *felis* FS-H members with *X*. *cheopis* sequences revealed varying degrees of sequence similarity (21.6% - 89.6%). Additionally, the phylogenetic analysis using the translated sequences of those CDS identified by mass spectrometry analysis resulted in the formation of four major clades (S3 Fig), similar to the one reported in the first *C*. *felis* sialome [21]. Clades I and II contained a mixture of both rat and cat flea sequences including the sodium channel blocker FS50. Clade III is composed of three *X*. *cheopis* sequences, including FS48, and all selected scorpion toxins, while clade IV contained only *C*. *felis* sequences. The low bootstrap values at the base of each clade in addition to the high abundance and variability between the FS-H members strongly suggest that this salivary protein family is under expansion, possibly resulting in proteins with different kinetic profiles. From the blood acquisition perspective, their presence in the flea saliva could ensure that different populations of channels are blocked during feeding, thus interfering with host nociception and immune signaling [49, 50]. However, considering the distant related clade IV (S3 Fig), we cannot discard the possibility that some FS-H-like proteins are acquiring new functions that are not related to their channel blocking activity, and future studies are required to uncover the role of the FS-H like proteins in blood acquisition and pathogen transmission. It’s noteworthy, that due to the high genomic variability between individual fleas, we cannot exclude the possibility that some of the CDS’s identified here are artefacts of our *de novo* assembly approach.

#### The histidine acid phosphatase superfamily

Acid phosphatases are enzymes widely distributed in plants and animals catalyzing the hydrolysis of phosphate monoesters in acidic conditions (pH 4–7) where two His residues have been shown to be essential for their catalytic mechanism. The first His residue is located in the RHG motif, which is conserved in all members of this protein family [51]. A small subset of this family are metallohydrolases, enzymes that present two metal ions in their catalytic center and are also known as purple acid phosphatases [52]. In addition to the flea sialomes, the acid phosphatases were also reported in the salivary glands of other hematophagous arthropods, including triatomines (males and females) [53] and ticks [54].

**Fig 2:**
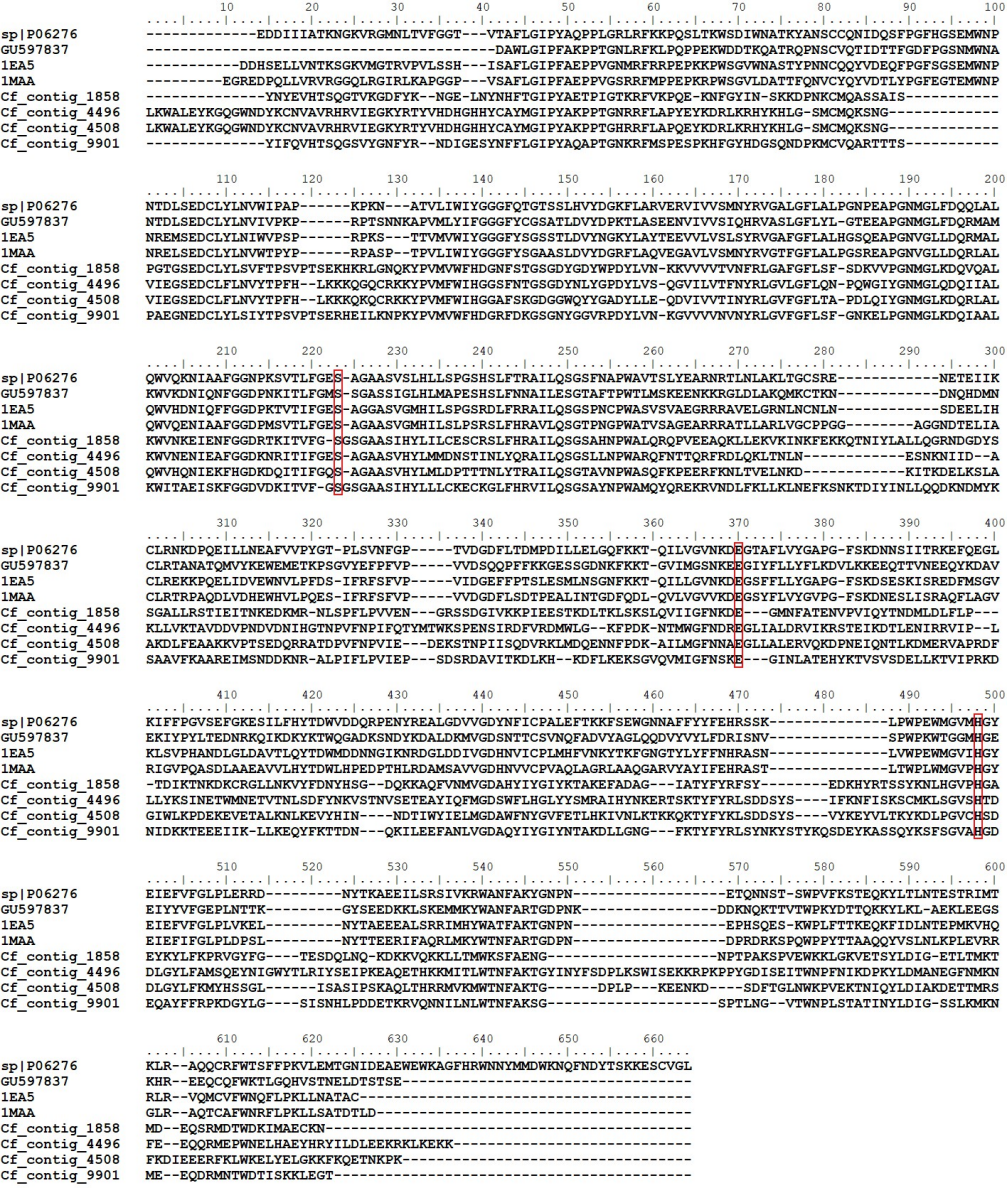
Amino acid alignment acid phosphatases. Human prostatic acid phosphatase (NP_001090.2) and acid phosphatases from *X*. *cheopis* (ABM55413.1, ABM55400.1, ABM55403.1, ABM55410.1, ABM55411.1, ABM55409.1) previously identified by mass spectrometry analysis in the flea salivary glands and *C*. *felis* acid phosphatases. The motifs containing the histidine residues relevant for the hydrolysis of phosphate monoesters are boxed in red.

In the previous *C*. *felis* sialome, 21 contigs classified as acid phosphatases and representing 17% of all secreted CDS’s were reported [21]. In our current dataset, we identified 35 CDS’s belonging to the acid phosphatase family with variable TPM values (2.67–14,955) (S1 File), and that together accounted for 15.6% of all secreted proteins. Supporting this data, our LC-MS analysis also identified unique peptides from 9 acid phosphatases that accounted for 24% of all secreted proteins (Table 2) and were the second most abundant salivary protein family from *C*. *felis*. Interestingly, all previously reported sequences lacked the catalytic His residue, suggesting that the expected catalytic activity is absent. In the current dataset, 34 out of the 35 CDS’s identified as acid phosphatases lacked the two His residues relevant to the monoester hydrolysis, while CDS contig_1524, found in moderate levels in the *C*. *felis* salivary glands (TPM = 11,070) (S1 File), encoded one of the two His residues (Fig 2). However, unique peptides from contig_1524 were not found in the proteome analysis (S2 File). Similar findings were also observed in the *X*. *cheopis* sialome, in which several CDS coding for acid phosphatases lacking the catalytic residues were found. Among those CDS, two putative acid phosphatases presented the catalytic motif RHG, although these CDS’s were not observed in the mass spectrometry analysis of *X*. *cheopis* salivary glands homogenates at different time points [37]. Therefore, it is not clear if the *expected* catalytic activity of acid phosphatases is present in the flea saliva.

From the blood-feeding perspective, it was originally proposed that the absence of residues associated with enzymatic activity of *X*. *cheopis* phosphatases might lead to the protein being permanently bound to its natural substrate, or acting as a kratagonist molecule [37], a sequestering molecule that limits its substrate’s availability [55]. This concept has been demonstrated in other blood-feeding arthropods (e.g., mosquitoes, ticks, sand flies and kissing bugs), in which a group of protein families was shown to bind to small agonists that play an important role in host homeostasis, including serotonin, epinephrine, histamine, nucleotides or eicosanoids [55]. In order to antagonize the hemostatic response triggered by such agonists it was speculated that the kratagonist must achieve a concentration between 0.2–2μM (the normal receptor saturating concentration of histamine, serotonin or ADP) [56]. In all cases reported so far, this agonist sequestering protein family is also the most abundant protein family in the blood feeder salivary glands [57–60]. Therefore, it is plausible that these *pseudo-acid phosphatases* from fleas have a similar role.

Finally, despite the overall low identity observed between *X*. *cheopis* and *C*. *felis* salivary acid phosphatases, the lack of the catalytic residues, their high abundance and variety in both flea species suggest that these *pseudo-enzymes* have a conserved role in the salivary glands of fleas.

#### Cholinesterase and esterases

Cholinesterases and esterases belong to the carboxylesterase family (PFAM 00135) and present a catalytic triad composed of Ser, Asp and His residues [61, 62]. Members of these families are frequently reported in sialome studies, and their catalytic activity was demonstrated in the saliva and salivary gland homogenates of different blood-feeding arthropods [63, 64]. In the first cat flea sialome three truncated esterase-like transcripts were reported [21]. Here we identified 25 CDS with TPM values from 1.13 to 3,036 with high sequence similarity to previously deposited cholinesterases and esterases (S1 File). Additionally, our mass spectrometry analysis of salivary gland homogenates identified unique peptides from six cholinesterase-like proteins, representing the 6^th^ most abundant protein family (Table 2) and confirming they are present in the salivary glands.

**Fig 3:**
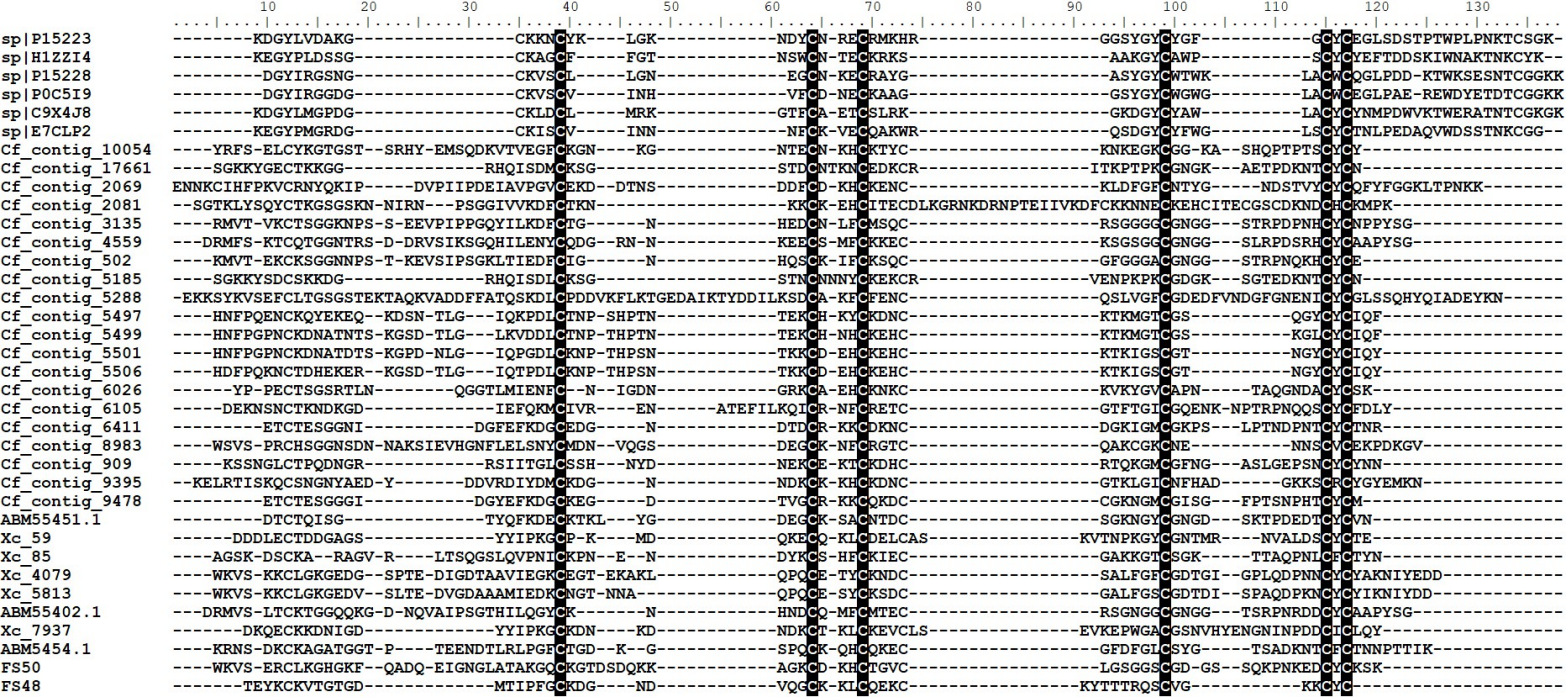
Amino acid alignment of *C*. *felis* cholinesterases. *C*. *felis* sequences found in the mass spectrometry analysis were aligned with sequences from *Torpedo californica* (1EA5), *Mus musculus* (1MAA), *Cimex lectularius* (GU597837) and the human (sp|P06276) cholinesterases. The catalytic triad residues Ser, Glu and His are red-boxed.

It is not clear if such enzymes play a role in blood acquisition. To date, only one salivary cholinesterase has been characterized. In the bed bug *Cimex lectularius* the salivary cholinesterase (ClSChe) was shown to be present in the arthropod salivary ducts, strongly suggesting that the protein is secreted into the saliva. Additionally, it was demonstrated that, although the catalytic residues are conserved in ClSChe (Fig 3), the recombinant enzyme presented low catalytic efficiency towards acetylcholine (Ach) [65], suggesting that ClSChe act as a chelator of Ach. Acetylcholine is traditionally known as a neurotransmitter; yet, Ach has also been shown to play a role in immunity and inflammation [66], which could interfere with blood acquisition. Finally, it’s important to note that Ach may not be the main target of these salivary cholinesterases. Therefore, future studies are necessary to uncover *C*. *felis* cholinesterases activity and physiological role.

#### Antigen 5

Antigen 5-like proteins, along with the Cysteine-rich secretory proteins and the Pathogenesis-related 1 proteins, compose the CAP superfamily. Members of this superfamily are commonly reported in different life forms [67]. They are the major components of vespids venom [68] and are considered toxins due to their ability to inhibit smooth muscle contraction [69]. The antigen 5-like proteins are commonly reported in sialome studies from blood-feeding arthropods [70–72], including the first *C*. *felis* sialome that reported two transcripts classified as antigen 5-like proteins [21]. Here we identified 15 CDS with high variability of TPM values (1.89–5,815), while LC-MS analysis identified the presence of unique peptides from 5 of them (Table 2).

From a functional perspective, only a handful of antigen 5-like proteins from hematophagous vectors have been characterized. In *Dipetalogaster maxima* and *Triatoma infestans*, antigen 5-like proteins show an antioxidant potential similar to superoxide dismutase, disrupting platelet aggregation induced by low concentrations of collagen [73]. In the horsefly *Tabanus yao* antigen 5-like proteins isolated from the salivary glands interfere with thrombus formation, angiogenesis and platelet aggregation [74, 75]. Interestingly, in mosquitoes, antigen 5-like molecules have been reported in the sialome of both males and females, even though some transcripts were 10- and 100-fold upregulated in females [38, 76]. Together these results suggest that members of this family have multiple roles in vector biology, including blood acquisition. Interestingly, the triatomine antigen 5 protein DMAV [77] has an alkaline pI, as do the female-enriched antigen 5 proteins of mosquitoes (but not the male-abundant antigen 5 proteins) [76, 78]. Of the 15 proteins identified as members of the Antigen 5 family, 14 have pI’s estimated at higher than 9.3, except for contig_14784, which has a pI of 5.8 and is the least expressed member of the family. It is tempting to speculate that the positive surface charge may act as a bait to increase local concentration of O_2_^**-**^ anions.

#### Apyrases

Apyrases are enzymes that catalyze the hydrolysis of ADP and ATP to AMP [79] and are ubiquitously found in hematophagous arthropods, including ticks [80], kissing bugs [81], mosquitoes [82, 83] and fleas [84, 85]. Apyrases are currently divided into three sub-families: the 5`-nucleotidase, *Cimex*-type and the CD-39 type [86]. It is worth noting that, among hematophagous vectors, apyrases from the CD-39 sub-family have only been reported in fleas [21, 43]. Functionally, apyrases have been shown to inhibit ADP-induced platelet aggregation [87, 88], facilitating blood acquisition. Additionally, phylogenetic analysis of apyrases from several blood-feeding vectors suggests that members of this protein family were selected independently through the course of a convergent evolution scenario [86], highlighting their importance for blood-feeding.

In the current dataset, we identified 10 transcripts classified as apyrase-like, 4 from the CD-39 sub-family and 6 belonging to the 5`-nucleotidase sub-family (S1 File). Our LC-MS analysis of salivary glands homogenates identified unique peptides from two CD-39 apyrases and one 5`-nucleotidase (S2 File). Similar to the findings of the rat flea, *X*. *cheopis* [37], both transcriptomic and proteomic analyses suggest that *C*. *felis* CD-39 apyrases are the most abundant apyrases in the cat flea and probably the main enzyme responsible for the degradation of ADP and ATP. It is possible that the flea salivary 5’-nucleotidase enzymes may function in the hydrolysis of AMP and adenine dinucleotides as was shown for the salivary 5’-nucleotidase of the sand fly *Lutzomyia longipalpis* [89].

#### Peptidase inhibitors

Peptidase inhibitors, mainly serine and cysteine peptidases, are ubiquitously reported in the sialome from hematophagous arthropods [11]. In our transcriptome analysis of *C*. *felis* salivary glands, we identified 8 transcripts annotated as serine peptidase inhibitors belonging to the Kazal, pacifastin, serpin and trypsin inhibitor-like (TIL) families with TPM values ranging from 2.2 to 4,866 (S1 File). In addition, our LC-MS analysis also revealed the presence of unique peptides from three of them (Table 2).

Salivary peptidase inhibitors from blood-feeding vectors are usually associated with the modulation of host immune response and homeostasis. As a rule of thumb, peptidase inhibitors that interfere with blood clotting usually do so by targeting factor Xa or thrombin, which are shared by both intrinsic and extrinsic pathways. In mosquitoes, it was demonstrated that the saliva of anophelines inhibits thrombin, while the saliva of culicine targets factor Xa [90]. Factor Xa and thrombin inhibitors were also reported in ticks [91], sand flies [92] and kissing bugs [93], suggesting that at least one anticoagulant molecule is present in the saliva of hematophagous arthropods. In fleas, only one thrombin inhibitor was recently characterized. XC-43 from the rat flea *Xenopsylla cheopis* inhibits thrombin with high affinity (Ki = 10 pM) and interferes with blood coagulation *in vitro* and *in vivo*. Additionally, the crystal structure of XC-43 complexed with thrombin reveals that XC-43 is not cleaved by the enzyme [19]. Interestingly, BLASTp of XC-43 against the *C*. *felis* genome and the current transcriptome dataset failed to produce significant *hits*, indicating that a similar molecule is absent in *C*. *felis* salivary gland homogenates. It’s possible that one of the three serine peptidase inhibitors identified in the LC-MS analysis is a thrombin or factor Xa inhibitor. Another possibility is the presence of a novel anticoagulant molecule in the cat flea salivary glands.

#### Odorant-binding protein (OBP)

OBP’s are proteins that usually display a hydrophobic binding pocket and are associated with the transport of small molecules related to insect chemoreception. Currently, insect OBP’s are classified into 4 sub-families based on their size and number of cysteine residues [94], and, structurally, they are constituted by a core structure composed of six α-helices that encloses the hydrophobic pocket [95]. In the current dataset, we identified six transcripts containing an OBP domain with relatively low TPM values (2–210) (S1 File), and unique peptides from only two of them (contig_45922 and contig_30935) were observed in our LC-MS analysis (S2 File). Both proteins were classified as a classical OBP containing 6 cysteine residues and a molecular weight near 14 kDa. The low overall abundance of these OBP’s is probably the reason why they were not identified in the previous *C*. *felis* sialome, which used a Sanger-based approach [21].

In addition to salivary glands, OBP’s are also commonly found in the insect’s sensory organs, where they have a key role in the insect’s ability to identify potential hosts, mating partners and food [96]. In the salivary gland, OBP’s role in blood-feeding is better understood in mosquitoes where several members of a multi-gene family named D7 have been functionally and structurally characterized. The D7 are the most abundant salivary protein of mosquitoes and function as *kratagonists*, binding to serotonin, epinephrine, histamine, and cysteinyl-leukotrienes [97, 98]. Histamine and serotonin dose response curves attain saturation levels at up to several micromolar for platelet aggregation or smooth muscle contraction assays. However, other agonists such as thromboxane A_2_ and leukotrienes are active at 10-fold smaller concentrations. Considering the low overall abundance of *C*. *felis* OBP’s, it is possible that they target these lipidic agonists.

#### Unknown proteins

Members of this protein “family” are sequences that we failed to classify and are simply named here as “unknown”. In our annotation strategy, we further subclassify *unknown* proteins into two groups: the *unknown conserved* class comprises sequences that have high similarity with previously deposited proteins of no known function, while the *unknown* class contains potential novel sequences since they have low or no sequence identity to deposited sequences. It’s important to note that because our sequences originated from a *de novo* assembly strategy, we cannot discard the possibility that some of the sequences under this “family” are artefacts of our assembly strategy and not true CDS, and, therefore, the data must be carefully interpreted.

In our analysis, we classified 108 putative secreted CDS in the *unknown* group, accounting for 33.5% of the TPM, while our LC-MS identified unique peptides from 13 of them, representing 11.6% of all identified proteins (Table 2). Additionally, 161 CDS (~7.9%) and 23 proteins (~13.8%) were classified as *unknown conserved* (Table 2). Together, they represent 41.4% of all secreted transcripts and ~25.4% of all secreted proteins (Table 2). These values are similar to those found in other sialomes [37, 38, 40, 76] and perhaps reflect a hidden pharmacological potential existing within the salivary glands of hematophagous vectors.

## Conclusion

Ten years ago, a Sanger-based sialome of *C*. *felis* reported 806 contigs, of which 91 encoded for putative secreted proteins, providing a glimpse into the cat flea salivary gland composition [21]. Here, using an Illumina-based RNA-seq approach, we reported 8,892 total CDS, of which 519 were annotated as potentially secreted proteins, providing a more comprehensive insight into *C*. *felis* salivary contents. Additionally, we paired our transcriptome study with a mass spectrometry analysis of salivary gland homogenates, confirming the presence of several putative secreted proteins in the tissue. Together, the data reported here represents an extended reference for future studies interested in characterizing *C*. *felis* salivary proteins and their potential role in blood acquisition.

### Future direction

With the advances in sequencing technologies, the full omics analysis (i.e., genome, transcriptome, and proteome) of a single flea has become a feasible task and will be a key effort to understand the underlying mechanisms responsible for such individual variation. The application of this individual omics approach to other hematophagous arthropods (e.g., ticks, mosquitoes, sand flies and kissing bugs) might also provide useful insights into the independent evolution of the hematophagous behavior.

## Supporting information

S1 Fig Spearman correlation of the RNAseq and proteomic relative quantifications. Scatter plot of the Log_2_NSAF by the Log_2_TPM **(A)** from all proteins identified by the LC-MS analysis and **(B)** only from the *matches* classified into the secreted functional class. A general linear model was fitted to the data (blue line) and the Spearman correlation was calculated.
(PDF)

S2 Fig Amino acid alignment of Cf-12 and contig_5185. Cf-12 was identified in the previous *C*. *felis* sialome and contig_5185 is from the current dataset. The putative signal peptide is underscored, the distinct residues between the two sequences are red-boxed and the cysteine residues are blue-boxed.
(PDF)

S3 Fig Phylogenetic tree of the FS-H-like proteins. Sequences from C. felis, *X*. *cheopis* and scorpion toxins were used and the tree was constructed using the Maximum likelihood model. The number at the bases of the branches represents the concordance between 500 bootstraps replicates.
(PDF)

S1 File The hyperlinked excel spreadsheet of the annotated CDS’s and the associated files. The file can be downloaded from the following link: https://proj-bip-prod-publicread.s3.amazonaws.com/transcriptome/C_felis/sialome_2022/cf.zip.
(TXT)

S2 File Excel spreadsheet containing the summarized results from the LC-MS analysis of *C*. *felis* salivary gland homogenates. The file can be downloaded from the following link: https://proj-bip-prod-publicread.s3.amazonaws.com/transcriptome/C_felis/sialome_2022/cf.zip.
(TXT)
