# Supplementary figures and images for: Revisiting the sialome of the cat flea Ctenocephalides felis
Source: PLoS One. 2023 Jan 17;18(1):e0279070. doi: 10.1371/journal.pone.0279070 (PMC9844850; doi:10.1371/journal.pone.0279070)

Supplementary figure 1:

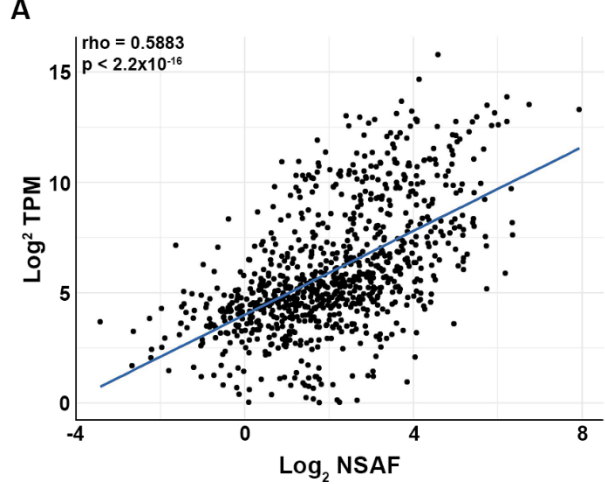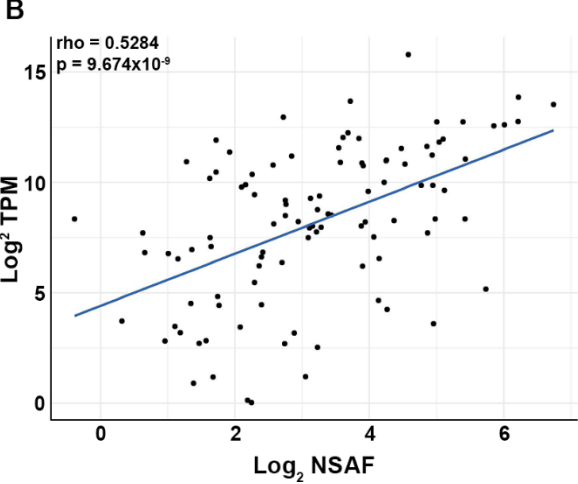

Supplement: S1 Fig — Scatter plot of the Log2NSAF by the Log2TPM (A) from all proteins identified by the LC-MS analysis and (B) only from the matches classified into the secreted functional class. A general linear model was fitted to the data (blue line) and the Spearman correlation was calculated. (PDF) [file pone.0279070.s001.pdf]

Supplementary figure 3:

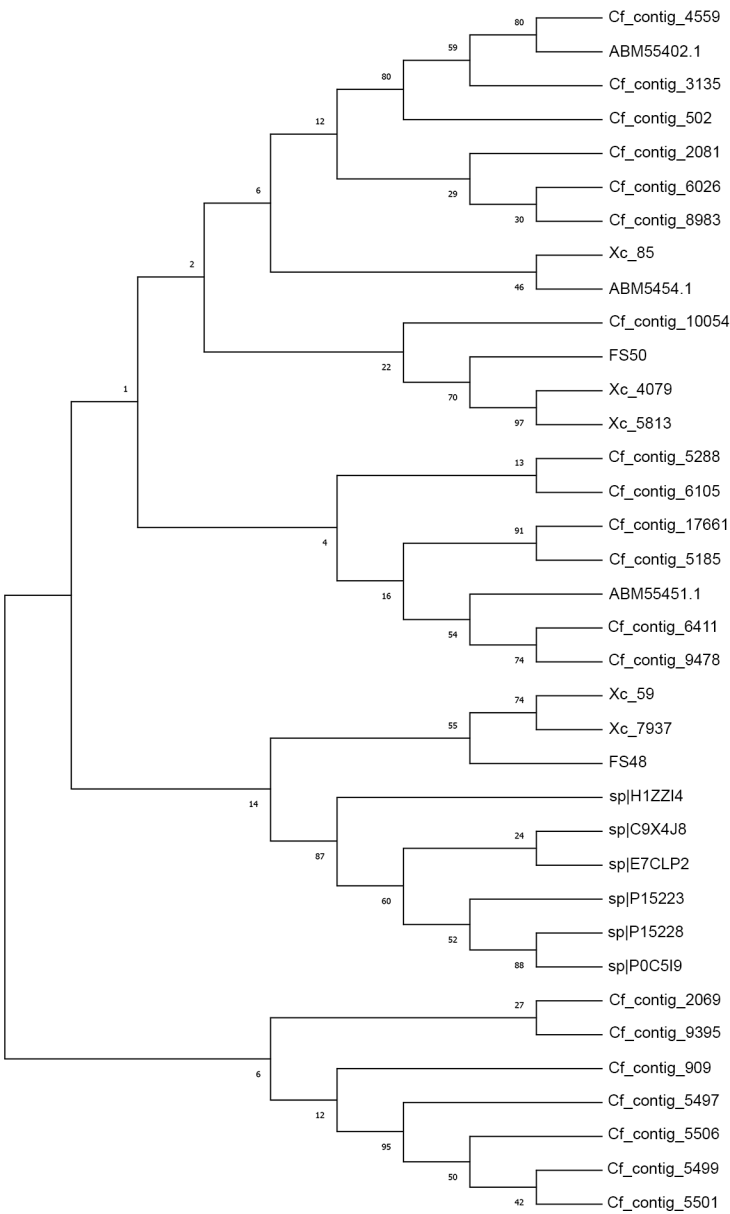

Supplement: S3 Fig — Sequences from C. felis, X. cheopis and scorpion toxins were used and the tree was constructed using the Maximum likelihood model. The number at the bases of the branches represents the concordance between 500 bootstraps replicates. (PDF) [file pone.0279070.s003.pdf]
