# Supplementary material for: Revisiting the sialome of the cat flea Ctenocephalides felis
Source: PLoS One. 2023 Jan 17;18(1):e0279070. doi: 10.1371/journal.pone.0279070 (PMC9844850; doi:10.1371/journal.pone.0279070)
Supplement: S2 Fig — Cf-12 was identified in the previous C. felis sialome and contig_5185 is from the current dataset. The putative signal peptide is underscored, the distinct residues between the two sequences are red-boxed and the cysteine residues are blue-boxed. (PDF) [file pone.0279070.s002.pdf]

Supplementary figure 2:

```
                         10        20        30        40        50        60        70        80
                ....|....|....|....|....|....|....|....|....|....|....|....|....|....|....|....|.
Cf-12           MKFWAILLIALVVVHCVFASGKQYSECSKKGGRHQISDLCKSGSTNCNNNYCKEKCRVENPKPKCGDGKSGTEDKNTCYCN
Cf_contig_5185  MKFWAILLIALVVVHCVFASGKKYSDCSKKDGRHQISDLCKSGSTNCNNNYCKEKCRVENPKPKCGDGKSGTEDKNTCYCN
```
